# Bioinformatics in Dentistry: Preoperative Healing Prediction Using Artificial Intelligence and Patient Blood Profiles in Electronic Health Records

**DOI:** 10.7759/cureus.104179

**Published:** 2026-02-24

**Authors:** Alhanouf A Alturki, Alanoud F Bin Muammar

**Affiliations:** 1 General Dentistry, Ministry of Defence, Riyadh, SAU; 2 Restorative Dentistry, Ministry of Health, Riyadh, SAU

**Keywords:** ai in dentistry, bioinformatics in dentistry, blood biomarkers, ehr blood profiles, healing prediction, machine learning models, oral surgery outcomes, platelet rich fibrin, precision dentistry, risk stratification

## Abstract

Bioinformatics and artificial intelligence (AI) have become a new direction in dentistry, enabling predictive, precision-focused surgical care. In oral surgery and dental implant procedures, postoperative healing outcomes are inconsistent across most cases. These outcomes are affected by systemic biological determinants that are often documented in electronic health records (EHRs) but are seldom used to forecast risk during preoperative care. This literature review focuses on the emerging role of AI-based predictive models that use blood biomarker profiles and nutritional information from EHRs to predict preoperative healing outcomes. Specific attention is paid to blood-based elements (platelet indices, leukocyte counts, fibrinogen levels, hemoglobin status, and micronutrient markers), which play critical roles in inflammation, angiogenesis, and tissue regeneration. The article also discusses how AI algorithms, such as machine learning and gradient-boosting methods, can use longitudinal EHR data to create personalized healing risk scores and automate the optimal utilization of autologous blood concentrates, including platelet-rich fibrin (PRF) and platelet-rich plasma (PRP). The results endorse a paradigm shift toward predictive, data-driven dentistry that maximizes natural healing prowess through digital innovation.

## Introduction and background

In dentistry, postoperative healing remains highly unpredictable and varies widely depending on surgical techniques and biomaterials. The clinical outcomes after procedures such as tooth extraction, ridge preservation, periodontal surgery, and implant placement are dependent not only on local surgical factors but also on systemic biological processes, such as immune status, hematologic balance, nutritional adequacy, and the burden of chronic illnesses. Conventionally, clinicians have used more generic risk factors, such as smoking status or diabetes, as opposed to personalized biological information when identifying their regenerative interventions. This disconnect prevents streamlining treatment plans and proactive screening of patients at risk of delayed or impaired recovery.

Bioinformatics and artificial intelligence (AI) offer a viable solution to this dilemma by facilitating the analysis and integration of high-dimensional, complex clinical data. Longitudinal data on lab values, demographics, medications, and comorbidities are directly related to tissue regeneration and wound healing and are stored in electronic health records (EHRs). Nonlinear relationships between these variables are also hidden and are not obvious to the human eye but can be discovered using machine learning (ML) algorithms [1]. Such models can be used to predict the trajectory of healing when implemented in preoperative settings, enabling personalized surgical planning.

**Figure 1:**
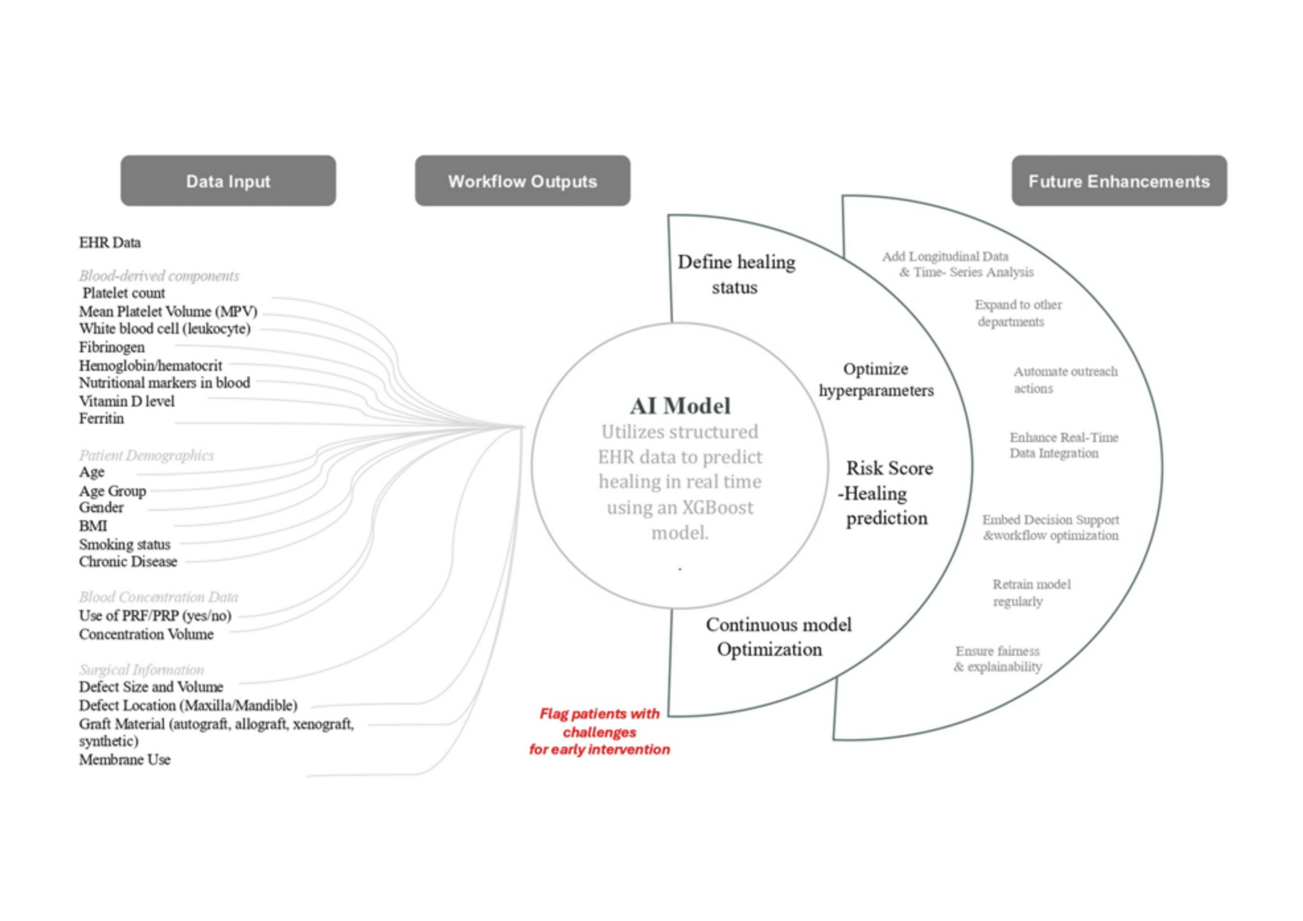
AI Model Design The image is the original work of the authors.

The application of autologous blood concentrates, such as platelet-rich plasma (PRP) and platelet-rich fibrin (PRF), has also attracted widespread interest in dentistry due to their ability to enhance angiogenesis, modulate inflammation, and promote bone regeneration. Nonetheless, the dissimilarity in blood composition among patients can vary considerably and affect the biological efficacy of these treatments. The integration of preoperative blood biomarker profiles into AI-based predictive models is a new paradigm in precision dentistry, enabling clinicians to design regenerative approaches based on each individual’s inherent healing capacity. Figure 1 presents a conceptual, proposed, EHR-derived blood biomarker integrative framework in machine learning-based predictive analytics of postoperative dental healing outcomes. The model is not meant to reflect a clinical system that is documented as validated and in use. XGBoost has been provided as an example because it is strong when using tabular clinical data, it can model nonlinear interactions, and it has previously been used in healthcare prediction tasks. Nonetheless, other algorithms, including random forests or deep neural networks, can be equally suitable based on the features of the data. No feature engineering, hyperparameter optimization, or validation strategy is implied, and empirical testing must be conducted before any clinical usage. The review analyzes existing evidence on the use of AI and bioinformatics to predict preoperative healing outcomes in dentistry using EHR-derived blood profiles.

## Review

Blood biomarkers and healing potential

Blood-based biomarkers play an essential role in wound healing and tissue regeneration. The key determinants of inflammation, angiogenesis, and collagen production are platelet indices and leukocyte subpopulations, fibrinogen, hemoglobin concentration, and micronutrients, including iron and vitamin D. Shome [2] emphasizes that the PRP and PRF effect is strongly contingent on platelet concentration, fibrin structure, and kinetics of cytokine release, factors that are determined by baseline hematologic parameters.

Autologous concentrates may therefore not be effective due to suboptimal regenerative capacity in patients with anemia, chronic inflammation, or nutritional deficiencies. Recent developments in patient blood management have underscored the importance of predictive modeling in assessing perioperative risks associated with the degree of hematologic imbalance. Coelho [3] shows that AI-based models, using routine blood tests, can predict transfusion requirements, postoperative complications, and recovery patterns. These nonlinear correlations cannot be readily identified through standard clinical evaluation but can be detected using machine learning techniques.

Artificial intelligence and EHR-based prediction

The use of AI on EHR data has evolved rapidly across all fields of medicine, including hematology, surgery, and regenerative medicine. Gradient boosting, random forests, and deep neural networks are machine learning methods that are effective for working with longitudinal data and identifying associations between clinical variables [4]. AI has shown promising predictive capabilities in wound-healing and surgical outcome research in the research phase, but dentistry-specific clinical validation is needed [5].

In dentistry, AI has been deployed for diagnostic imaging, caries detection, periodontal risk assessment, and treatment planning. According to Ghods [6], clinical decision support systems enhanced by AI improve the accuracy and consistency of diagnostic results and reduce the workload of clinics. In more recent times, there has been discussion of the application of AI in incorporating systemic health data into dental risk measures.

Gaddam [7] demonstrates that machine learning can analyze the potential complexities of an immunological response in periodontal disease, highlighting the applicability of systemic biomarkers to oral health outcomes.

Bioinformatics in regenerative dentistry

Bioinformatics provides the theoretical foundation for AI-based analysis of biological and clinical data. Bioinformatics tools in regenerative dentistry help combine molecular biology, hematologic data, and clinical outcomes to enable precision-based interventions. As Saberian [8] indicates, AI-based regenerative solutions can streamline stem cell therapy, scaffold development, and growth factor delivery using patient-specific biological information. Similarly, Nosrati and Nosrati [9] and Abuarqoub and Mutahar [10] discuss the potential of AI as a transformative factor in regenerative medicine, but they also raise issues of data quality, algorithmic transparency, and ethics. Surgical disciplines have demonstrated the potential of preoperative planning aided by AI, enabling individualized risk stratification and more accurate prediction of outcomes [11]. It is logical and effective to apply these solutions to dentistry through blood profiling in EHR systems.

Integration of systemic health data into dental predictive models

The incorporation of systemic health information into dental decision-making is a very important milestone in precision dentistry. Modern studies focus on the idea that the results of oral healing are highly determined by the systemic physiological state, such as inflammatory state, immune functioning, and hematologic balance. The British Dental Journal notes that the use of artificial intelligence (AI) has facilitated oral medicine in going beyond localized measurements and integrating more general patient health information into the predictive framework of clinical practice [12]. This change is significant in preoperative dental practice, in which systemic variables have the power to impact postoperative recovery and regenerative outcomes.

EHRs offer a universal platform to incorporate blood biomarkers, including leukocyte profiles, hemoglobin levels, and inflammatory indices, into AI predictive models. This can be achieved through bioinformatics tools that standardize heterogeneous sources of data and extract features from longitudinal patient records. AI-based models are thus able to detect complicated interactions between systemic biomarkers and oral tissue responses that cannot be readily determined using conventional risk assessment [13]. Periodontal research evidence shows that the inclusion of systemic indicators significantly enhances the predictive abilities of AI models compared to models based only on clinical dental parameters [14]. With the integration of these predictive systems into the EHR infrastructure, dental practitioners can be provided with personalized healing predictions before undergoing a surgical procedure. This method facilitates proactive treatment planning, a risk-differentiated approach, and customized regenerative strategies.

AI-based risk stratification for periodontal and surgical outcomes

Risk stratification is an essential element of planning surgery, and modern dental assessment instruments do not usually take into consideration systemic biological differences amongst patients. Artificial intelligence has become a strong tool to overcome this drawback by providing data-based classification of patients according to personal risk profiles. Roy [14] demonstrates a high level of accuracy in machine learning models in forecasting the severity of periodontal disease, its development, and the outcome of treatment, especially in cases where biological and systemic features are also used as part of the analysis framework.

These findings have direct implications for preoperative oral surgery. Biomarkers in blood derived from immune competence and inflammatory burden that are known to be causative factors in wound healing and regenerative response can be used in AI-based risk stratification models. Clinicians are able to adjust the timing of surgery, choose adjunctive regenerative therapies, or increase the intensity of postoperative surveillance by detecting patients who are more prone to delayed healing or postoperative complications. The British Dental Journal also emphasizes that AI-enhanced clinical decision support systems enhance consistency and reduce subjectivity of treatment planning in the oral healthcare setting [15]. Notably, the integration of AI-powered risk stratification systems into EHR systems will guarantee their availability in real time and clinical viability. This form of integration has enabled dental professionals to take advantage of already gathered blood information without adding to the burden on patients.

Ethical, interpretability, and data quality considerations in dental bioinformatics

EHR-generated datasets can include missing data, inconsistencies in documentation, and institutional factors that can negatively impact model performance and generalizability. The first issue is data quality, since datasets obtained through EHR often lack values, have inconsistencies, and bias in documentation. The Diagnostics review highlights that insufficient preprocessing and validation may affect the reliability of models as well as restrict extrapolation to patient groups [16]. Normalization, feature selection, and cross-validation are thus useful bioinformatics techniques that contribute to sound predictive capability.

Another severe concern is model interpretability, especially when using it in a clinical setting where informed decision-making is required. The British Dental Journal emphasizes that explainable AI plays a crucial role in oral medicine, as it is essential to preserve clinician trust and the possibility of communicating with patients about the risks and outcomes of treatment [17]. Even though black-box models can be accurate, they might not be clinically adopted, as the decision logic of such models cannot be sufficiently explained. Ethical issues also extend to patient privacy and equity [18]. EHR-linked blood data create problems of data security and consent, particularly with the increasing interconnection of AI systems across healthcare sectors.

Multimodal data fusion in AI-driven dental prediction

There is growing research evidence that suggests that predictive accuracy in healthcare would be enhanced when multiple data modalities are combined for analysis, as opposed to analyzing them separately. Multimodal data fusion in dentistry is an approach that entails incorporating hematologic biomarkers, clinical parameters, radiographic data, and demographic data and integrating them into AI-based frameworks. Feher [18] emphasizes that structured EHR data, with the addition of biological markers, allow more accurate prognostic predictions than single-source datasets. This method is specifically applicable to regenerative dentistry, in which recovery of tissues depends upon local tissue factors as well as general physiological health.

Ensemble models and deep neural networks are machine learning architectures that are best adapted to modeling multimodal inputs because of their ability to model nonlinear interactions involving variables [9]. It is possible to use AI systems to produce personalized estimates of healing time, risk of complications, and achievement of regeneration, which are compared with clinical results in the oral cavity. These features facilitate the shift to personalized dental treatment and help strengthen the viability of EHR-based predictive dentistry, as presented in Table 1.

Role of inflammatory and immune pathways in oral regeneration

Inflammation and immune regulation are biological processes that play an important role in wound healing and tissue regeneration. The systemic outcomes of inflammatory load, as reflected by leukocyte counts, neutrophil-lymphocyte ratios, and acute-phase inflammatory products, have been proven to influence healing outcomes following surgery. According to Zhu [5], AI models that use inflammatory biomarkers can predict wound healing better than those that are reliant on clinical observations only.

In implant and periodontal dentistry, immunodulators are correlated with failure to heal, high risk of infection, and impaired regeneration. Gaddam [7] confirms that machine learning methods are able to work with the complexity of immune aspects in the development of periodontal diseases. By incorporating these immune and inflammatory biomarkers into AI-based dental prediction systems, these systems are reinforced and become more effective in terms of biological significance and predictability for patients with a higher risk of poor regenerative therapeutic results.

Predictive analytics for timing and selection of regenerative interventions

In addition to risk stratification, AI-based predictive analytics can be of clinical value in establishing the most appropriate timing and choice of regenerative interventions. Blood biomarker effects suggest that a patient is medically ready to undergo surgery, or the patient might need to undergo systemic optimization before surgery. As demonstrated by Coelho [3], AI-based patient blood management systems have the potential to predict recovery curves based on standard laboratory measurements, indicating that they can be paralleled in dental surgery.

Regarding predictive insights in regenerative dentistry, the choice to use PRP, PRF, bone grafting material, or a scaffold-based treatment approach can be made based on these predictive insights. Saberian [8] highlights that AI-informed bioinformatics platforms may help direct customization of regenerative approaches with reference to the specific biological traits of an individual. The use of predictive analytics in preoperative planning helps optimize resource allocation and increase the likelihood of positive patient outcomes.

Clinical translation and workflow integration in dental practice

Although the theoretical advantages of AI-friendly bioinformatics have already been recognized, practical clinical use can only be achieved through smooth integration of AI technology into current dental procedures. Modifications of EHR with decision support systems enable clinicians not to be burdened by administrative challenges, thereby facilitating the provision of predictive insights in real time. One of the most effective ways AI-based solutions can succeed in the oral health field is to act more as assistive tools than as authoritative systems in clinical decision-making. Kowal [11] also proves that AI-enhanced preoperative planning can play a role in enhancing the efficiency and consistency of workflow in various fields of surgery. In dentistry, blood-based predictive models are integrated into the EHR system, which allows clinicians to obtain individual predictions of healing during a regular clinical visit. This practical relevance is a strength that will facilitate adoption and correlates with the clinical implications of the studies presented in Table 1.

Implications for precision dentistry and future research

The growth of AI-based bioinformatics in dentistry is an indicator of a more general move toward precision dentistry, with prevention, treatment, and regeneration customized to the biological profile of individuals. According to Rokaya [16], AI-based predictive modeling is one of the essential parts of the future dental care system, especially with the advent of more data and greater compute capabilities.

Future studies need to focus on prospective biomarker selection. Explainable AI frameworks that can be used in routine clinical environments should be developed, and predictive models derived from EHR should be validated. Continued interdisciplinary cooperation among dentistry, hematology, and data science will be necessary to realize the full potential of predictive regenerative dentistry.

Synthesis of evidence

Collectively, the studies summarized in Table 1 demonstrate that integrating AI, blood biomarkers, and bioinformatics within EHR systems provides a scientifically grounded pathway toward predictive and personalized regenerative dentistry, supporting improved surgical planning, risk stratification, and healing outcomes across dental specialties.

**Table 1:** Summary of Key Findings From the Review AI: artificial intelligence, PRP: platelet-rich plasma, PRF: platelet-rich fibrin, ML: machine learning, HIV: human immunodeficiency virus, EHR: electronic health record.

Study / Source	Focus Area	Key Findings	Relevance to Dentistry
Thomas et al. (2025) [1]	Artificial intelligence in modern clinical practice	Integrating AI into busy healthcare systems can be difficult and costly	Stresses on the use of AI in modern healthcare practices
Shome et al. (2024) [2]	PRP/PRF biology	Platelet concentration and cytokine profiles influence regenerative efficacy	Highlights the need for individualized blood profiling
Coelho et al. (2025) [3]	AI in patient blood management	AI predicts recovery trajectories using routine blood tests	Applicable to predicting dental surgical healing
Nazha et al., 2025 [4]	AI in hematology	AI automates blood diagnostics, predicts treatment outcomes, and addresses clinical bias	Informs oral surgery planning, bleeding risk, and PRP/PRF optimization
Zhu et al. (2025) [5]	AI in wound healing	AI models accurately forecast wound healing outcomes	Reinforces the feasibility of AI- based healing prediction
Ghods et al. (2023) [6]	AI in clinical dentistry	AI improves diagnostic accuracy and decision-making	Supports expansion into predictive healing models
Gaddam et al., 2025 [7]	AI in periodontal disease & HIV	AI predicts periodontal progression and integrates systemic biomarkers for risk assessment	Improves periodontal diagnosis and links oral health with systemic conditions
Saberian et al. (2024) [8]	AI in regenerative dentistry	AI optimizes regenerative therapies using biological data	Aligns with precision-based dental regeneration
Kowal et al. (2025) [11]	Preoperative planning	AI enhances surgical planning and risk stratification	Relevant for dental implant and surgical planning
Nosrati C Nosrati (2023) [9]	AI in regenerative medicine	Bioinformatics enables the translation of biological data into clinical practice	Foundation for predictive regenerative dentistry
Jafer et al. (2025) [13]	AI in periodontal prediction	ML models predict disease and outcomes using biological data	Supports systemic biomarker integration
Alotaibi & Deligianni (2024) [12]	AI in oral medicine	AI integrates systemic health into dental decision-making	Reinforces EHR-based predictive dentistry
Roy et al. (2025) [14]	Artificial intelligence applications in periodontal diagnosis	Artificial intelligence models, such as machine learning and deep learning algorithms, proved to be highly accurate in the process of diagnosing the degree of periodontal disease through the analysis of clinical parameters, radiographs, and patient information. The article focuses on the combination of AI and digital health records to improve the process of early detection and risk stratification	Provides evidence of the viability of this addition to dental EHR systems: AI-based predictive models can be utilized to predict preoperative risks and personalized information on healing time and success, depending on systemic and oral health data
Mallineni et al. (2024) [15]	AI-driven wound healing prediction using biological and clinical data	A combination of blood biomarkers, inflammatory markers, and clinical profiles of patients, AI, and bioinformatics- based models was effective in predicting wound healing outcomes. The research also emphasizes the role of multidimensional integration of data in proper prediction of healing and treatment planning	Strengthens the relevance of bioinformatics and blood- based predictive analytics to dental surgery, specifically predictive analytics to determine postoperative healing outcomes based on preoperative blood profiles in EHR
Rokaya et al. (2024) [16]	AI in dentistry and biomaterials	AI supports diagnosis, treatment planning, and outcome prediction across dental specialties	Broadens AI applicability in dental care and predictive modeling
Lu et al. (2025) [17]	ML in regenerative endodontics	Random Forest and Gradient Boosting models effectively predict healing outcomes using clinical variables	Demonstrates AI-driven prognosis prediction in dental healing
Feher et al. (2024) [18]	AI data modalities in dentistry	Prognostic modeling using structured data enables personalized risk prediction in oral health	Supports integration of EHR and multimodal data for healing prediction
Abuarqoub & Mutahar, 2025 [10]	AI in regenerative medicine	AI enhances stem cell characterization, scaffold design, and tissue engineering	Supports dental bone grafts, stem cell therapies, and personalized implants

Discussion

This review shows that bioinformatics and artificial intelligence can be used to improve regenerative dentistry by predicting healing outcomes preoperatively using EHR-derived blood biomarkers. By integrating hematologic, inflammatory, and nutritional parameters, AI models can detect intricate biological patterns that may influence tissue regeneration and postoperative recovery [19]. Such a strategy facilitates the shift to less generalized and more biologically informed treatment planning. AI-based prediction plays a specific role in optimizing autologous blood concentrates such as PRP and PRF, whose clinical performance depends on the specific composition of blood. Preoperative screening of patients with suboptimal regenerative capacity could enhance patient selection, optimize treatment regimens, and decrease postoperative complications [20]. Also, longitudinal EHR data enable continuous model learning, facilitating adaptive clinical decision-making.

Common measures of clinical AI models include area under the receiver operating characteristic curve (AUC), sensitivity, specificity, positive predictive value, and negative predictive value. Nevertheless, these measures are not always reported in the dental AI literature, which makes comparison of studies difficult. XGBoost has the benefit of using gradient-boosting techniques that can be applied to structured clinical data, whereas deep learning models might necessitate significantly larger sample sizes. There is no agreement to date on the best modeling method for predicting dental healing outcomes after operations.

Altogether, AI-based bioinformatics may provide a framework to enhance predictability, efficiency, and outcomes in regenerative dental procedures and supplement clinician expertise. These models can become part of the standard preoperative oral examination as digital health systems develop.

Limitations

The findings of this review have several limitations that should be taken into account when interpreting them. The supporting evidence is based on medical and surgical fields, not necessarily on dental clinical trials. Thus, it cannot be directly applicable to oral tissues and procedures. The reliability of AI-based predictions may also be affected by differences in the quality of EHR data, including missing values, different laboratory measurement standards, and institutional differences [20]. In addition, not every machine learning model can be interpreted, which may reduce clinician trust and restrict adoption. There are also ethical concerns related to patient data privacy, biases in algorithms, and access to AI technologies, which make implementation more difficult [19]. Finally, the review is not founded on validated clinical outcomes but on predictive feasibility. Future dental research focused on standardized biomarker panels and explainable AI models is required to guarantee clinical effectiveness and safe integration into routine dental practice. Responsible implementation will also require regulatory guidance and training of clinicians.

## Conclusions

The introduction of bioinformatics and artificial intelligence into dentistry is a crucial transition toward predictive, personalized, and biologically informed care. Regenerative medicine, hematology, and wound-healing studies show that blood biomarkers are effective predictors of tissue healing potential and surgical success. Accurate preoperative prediction of healing trajectories can be supported by these biomarkers when analyzed using AI-driven models based on EHR data.

Such predictive capabilities in regenerative dentistry have major implications for optimization of autologous blood concentrates such as PRP and PRF, patient selection, and reduction of postoperative complications. However, issues concerning data standardization, validation, and ethical implementation should be considered.
